# Quantification of Isoflurane Uptake for Immobilization of Ostrich Embryos for Preclinical In Ovo Imaging

**DOI:** 10.3390/life16030525

**Published:** 2026-03-22

**Authors:** Thomas Winkens, Wiebke Neuschulz, Hans-Wolfgang Hoppe, Olga Perkas, Philipp Seifert, Falk Gühne, Julia Greiser, Martin Freesmeyer, Christian Kühnel

**Affiliations:** 1 Helios Clinic Erfurt, University Campus of the Health and Medical University, 99089 Erfurt, Germany; 2 Translational Nuclear Medicine and Radiopharmacy, Clinic of Nuclear Medicine, Jena University Hospital, 07747 Jena, Germany; 3 Clinic of Nuclear Medicine, Jena University Hospital, 07747 Jena, Germany; 4 MVZ Medizinisches Labor Bremen GmbH, 28357 Bremen, Germany

**Keywords:** isoflurane, in ovo imaging, ostrich egg, animal model, alternative animal testing

## Abstract

Preclinical imaging has recently been expanded through the use of ostrich embryos as an alternative in vivo model. In ovo experiments represent a promising substitute for conventional rodent-based animal testing. For artifact-free dynamic nuclear medicine imaging, reliable immobilization of embryos is essential. Although previous studies have demonstrated the feasibility of isoflurane anesthesia, the kinetics and uptake mechanisms of isoflurane in ostrich embryos remain insufficiently characterized. The aim of this study was to characterize gas exchange dynamics in ostrich eggs and to quantify isoflurane uptake using two complementary approaches: indirect consumption measurements in a closed system and direct quantification by serial blood sampling. Fourteen ostrich eggs, including seven fertilized and seven unfertilized specimens, were analyzed at developmental stages up to day 37 of incubation. Gas exchange was assessed in a sealed container using a clinical anesthesia gas monitoring system to measure oxygen consumption and carbon dioxide excretion. Isoflurane uptake was evaluated during exposure to concentrations of 2%, 4%, or 6%. In a separate experimental series, serial blood samples were collected during and after exposure to the same concentrations to determine systemic uptake. Fertilized embryos showed progressive increases in metabolic activity, with a maximal oxygen consumption and carbon dioxide excretion of 116 mL/h/kg and 93 mL/h/kg on day 37. Indirect measurements demonstrated isoflurane uptake rates of up to 1.1 mL/min at 6%, with proportionally lower values at 4% and 2%. Blood analyses confirmed systemic absorption, peak concentrations of 160 µg/mL, and detectable residual levels for up to 120 min after exposure. These findings refine in ovo imaging.

## 1. Introduction

Recently, preclinical imaging has been augmented by a relatively new concept: using ostrich (*Struthio camelus*) embryos for in vivo experiments, which are carried out in ovo. This concept represents a potential alternative to animal testing using rats or mice [1,2,3,4], as research using eggs does not fulfill the criteria of animal testing as long as all experiments are carried out before the egg hatches [5,6,7,8].

Preclinical imaging plays an important role in nuclear medicine because the development of new radiopharmaceutical compounds requires in vivo testing for assessment of the compounds’ biodistribution. Commonly, rats and mice are used for in vivo imaging studies of radiopharmaceuticals; however, it was hypothesized that in ovo imaging using ostrich eggs could serve as a pre-selection tool in order to identify compounds with promising biodistribution first, followed by verification studies in rodents. It should be noted that metabolic pathways in avian embryos may differ from those in mammals. Therefore, the in ovo model is intended as an early screening tool to identify promising biodistribution patterns, while detailed pharmacokinetic evaluation still requires validation in a smaller number of rodent models. Ostrich models require specialized equipment (estimated one-time hardware costs are approximately 6000–9000 €), but do not involve the breeding of colonies or continuous animal housing. Consequently, they avoid the husbandry costs associated with conventional rodent vivaria. Furthermore, in ovo research complies with the principles of modern animal testing (3 R) as established by Russel and Burch in 1959 [9].

Apart from sparing rodents from use in animal experiments, the use of large ostrich eggs (instead of chicken eggs, which are substantially smaller) allows for the use of imaging devices commonly used in routine clinical examinations in humans (e.g., computed tomography, CT; magnetic resonance imaging, MRI; and positron emission tomography, PET). In ovo imaging of chicken eggs requires dedicated small animal imaging devices, which represent a disadvantage regarding limited access, cost, and space [1,2,4].

Preclinical in vivo imaging requires immobilization in order to produce artifact-free imaging [10,11]. It has to be emphasized that immobilization is a crucial factor in nuclear medicine imaging, i.e., dynamic PET experiments, assessing in vivo biodistribution of radiopharmaceuticals over time. Animal motion significantly hampers correct quantification of biodistribution in different organs. In rodents, this problem is addressed by continuous isoflurane anesthesia [12]. Previous studies have described the use of isoflurane for successful immobilization of ostrich embryos [1,13]. However, there are no studies that elucidate the process of isoflurane uptake.

Thus, this study aims to (a) investigate gas exchange in ostrich eggs, (b) quantify the isoflurane consumption of ostrich eggs containing developing embryos and (c) verify isoflurane uptake by serial blood sampling and quantification of serum isoflurane concentration over time.

## 2. Materials and Methods

### 2.1. Ostrich Eggs

Ostrich eggs were collected once per week from a local ostrich farm located approximately 15 km from the imaging facility between April and September. The farm held seven flocks, each consisting of one rooster and up to five hens; thus, fertilization of eggs was natural. All eggs that were laid during one week were stored at room temperature and collectively transported by car, with separate holders for each egg. Due to the short transport time, no additional temperature control measures were applied. Artificial incubation was started 1–4 days after laying and was carried out using a multistage egg incubator (Sofie 3, Hemel, Verl, Germany) with constant incubation properties at 36.5 °C and 25% air humidity, as described elsewhere [2,4]. Non-fertilized eggs and fertilized eggs, as well as embryo viability, were classified by means of CT imaging (developing embryo), candling (growing shadow) and magnetovography (heartbeat) at different time points (development days, DDs 9–37) [1,2,14]. Eggs containing dead embryos were discarded in order to reduce the spread of bacterial infection to viable eggs.

If artificially incubated, ostrich eggs usually hatch after 42 days [15]. As it was a requirement to end all experiments before hatching, studies were performed on or before DD 37. This embryo study did not qualify as an animal research study according to the Federal German Animal Protection Act. The European Directive 2010/63/EU on the protection of animals used for scientific purposes and the Animals (Scientific Procedures) Act exempts bird embryos from their scope. Registration took place with the Office for Consumer Protection of the Thuringia State, registration number 22-2684-04-02-114/16. Thus, ethics committee approval was waived.

### 2.2. Egg Shell Composition, Physiology of Gas Exchange

During ostrich embryo development, the uptake and elimination of anesthetic gases follow the maturation of gas-exchanging organs, paralleling the well-characterized stages of the chicken embryo as described by Hamburger and Hamilton [16]. Given the similar developmental patterns of precocial birds and the fact that the ostrich’s incubation time is twice that of the chicken, a chicken model serves as a valid reference [15,17]. In ostrich embryos, organogenesis is predominantly completed on DD 18, after which development is characterized by embryonal growth and maturation [15]. The embryonic period can be divided into an early phase of organogenesis and a later phase dominated by growth [17]. The cardiovascular system, derived from mesodermal germ layers, begins functioning within 30 h of development as a primitive heart tube, which progressively differentiates into atria and ventricles [18,19]. In parallel, extraembryonic structures such as the chorioallantoic membrane (CAM), formed by fusion of the chorion and allantois, play a pivotal role in respiratory gas exchange, reaching maximal expansion around DD 12 [18,20]. Oxygen diffuses through eggshell pores and shell membranes into the CAM vasculature, with diffusion initially sufficient but later complemented by cardiovascular perfusion [20,21]. Increased metabolic demands lead to vascular remodeling and blood flow adaptations, although hypoxia and hypercapnia progressively develop prior to internal pipping [19]. After internal pipping, which is triggered by elevated CO_2_ levels [22], pulmonary respiration takes over, marking a major physiological transition.

### 2.3. Experiment Setup

**Figure 1:**
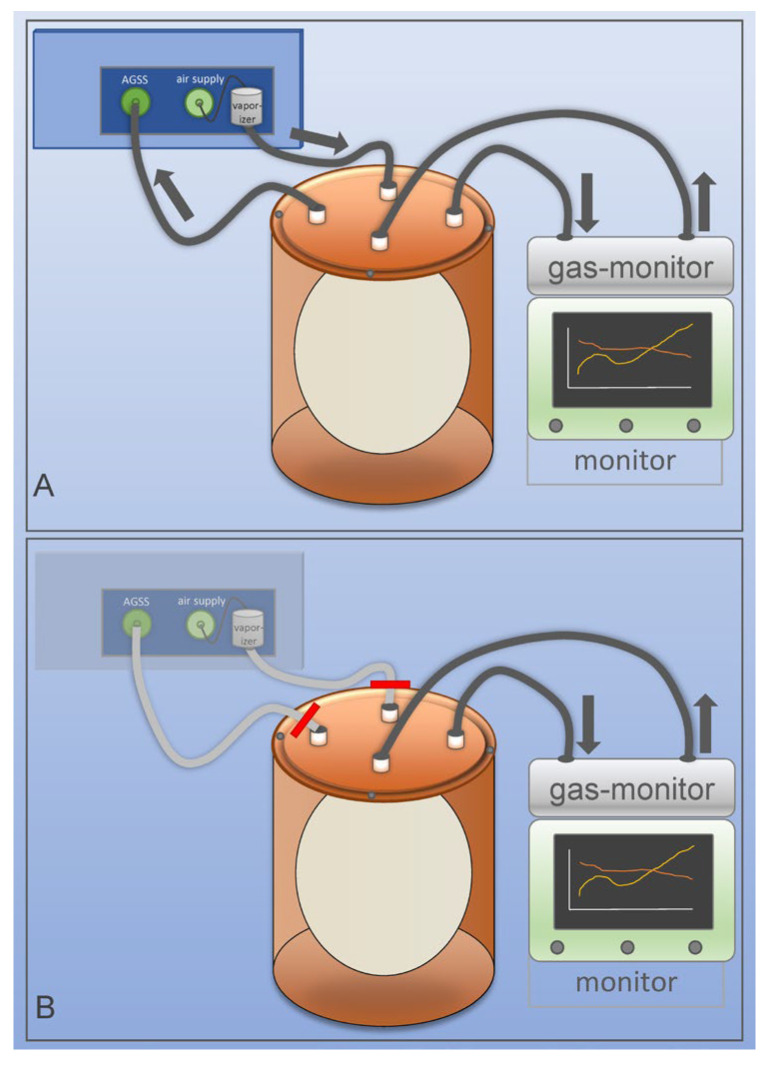
Experimental setup. (**A**) Modified airtight container based on a clinical CO_2_ absorber canister, connected to a isoflurane vaporizer and gas monitoring system. (**B**) Fully sealed system allowing continuous measurement of isoflurane concentrations and CO_2_ and O_2_ parameters in ambient air. AGGS: anesthetic gas scavenging system.

To indirectly assess anesthetic gas uptake in ostrich eggs, decreases in isoflurane concentration in the ambient air during exposure were monitored. A modified container based on a commercially available CO_2_ absorber canister (Dräger M29994, Drägerwerk AG & Co. KGaA, Lübeck, Germany) of an anesthetic unit (Fabius GS, Dräger) was used due to its gas-tight properties. The container, with a volume of approximately 2470 mL, was equipped with four ports for gas inflow and outflow in the lid, allowing controlled exposure to air and anesthetic gases (Figure 1A). Isoflurane gas was produced using a vaporizer, and gas-tight tubes connected the vaporizer and container. Excess gases were safely removed through an anesthetic gas scavenging system (AGSS) to prevent ambient contamination. The container was filled with isoflurane until the desired isoflurane gas concentration was achieved. Continuous monitoring (Gasbank, Scio Oxi Four and Infinity Gamma XL, Drägerwerk AG & Co. KGaA, Lübeck, Schleswig-Holstein, Germany) confirmed that the concentration was correct, and subsequently, the system was sealed with stopcocks to maintain a closed environment (Figure 1B). The gas concentrations of isoflurane, CO_2_, and O_2_ were then continuously recorded over 120 min or until a predefined oxygen drop from 21% to 15% occurred (in order to prevent embryonal damage by hypoxia, as previous experiments have shown increased embryonal mortality at lower oxygen levels). The entire setup was maintained at a stable temperature of 35.0 °C within a neonatal incubator (Isolette C2000, Dräger) to ensure physiological conditions. Prior to the experiments, a leakage test was conducted to ensure the integrity of the system.

### 2.4. Experiments

#### 2.4.1. Oximetry and Capnometry

CO_2_ and O_2_ were assessed in serial measurements in seven ostrich embryos on DDs 1, 4, 7, 10, 13, 16, 19, 22, 25, 28, 31, 34 and 37. Using the eggs’ volume and weight, the container volume, and the CO_2_ and O_2_ concentrations as determined by the gas monitor and standard conditions (temperature 35 °C and air pressure 1.0 bar), absolute excretion/absorption rates were calculated and expressed as mL/h/kg. Oximetry and capnometry aimed to verify the experimental setup, comparing the acquired data to already published data by Gefen and Ar [17]. Data were corrected for minor constant leakage effects. Isoflurane was not used in the referenced experiment.

#### 2.4.2. Isoflurane

Indirect isoflurane absorption was determined for three different concentrations (2%, 4%, 6%) on DD 34. Four ostrich embryos were investigated in each group. The setup was similar to the oximetry/capnometry setup; however, we filled the container with an air/isoflurane gas before sealing the container. After sealing, the experiment was continued until stop criteria were reached (i.e., oxygen < 15%). In this experiment, data were also corrected for minor constant leakage effects. Data were expressed as Vol.% and mL/min.

#### 2.4.3. Blood Sampling

**Figure 2:**
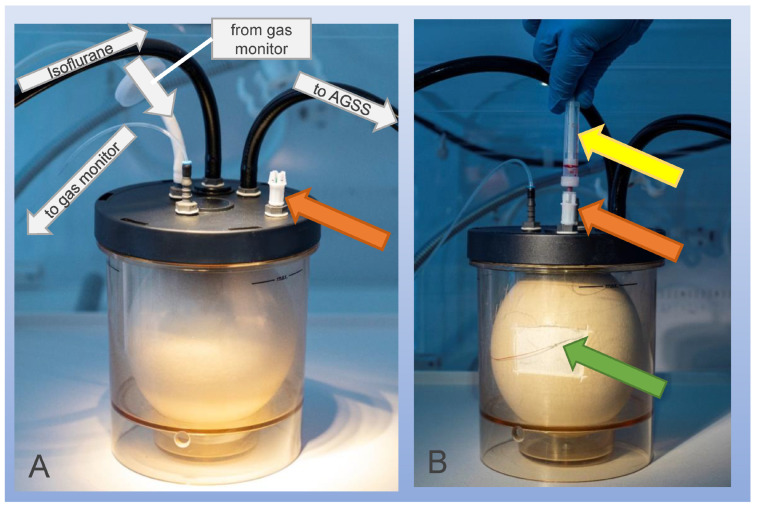
Experimental setup for direct collection of 500 µL blood samples at 1 min, 40 min, 58 min, 65 min, 90 min and 120 min after the experiment’s start. (**A**) shows a photograph comparable to Figure 1A; the orange arrow marks the adapter to connect a tube for blood tests. (**B**) shows the tube for blood tests (yellow arrow) and the needle (green arrow) after puncture of a chorio-allantois-membrane vessel. AGGS: anesthetic gas scavenging system.

Direct isoflurane uptake was determined using serial blood samples for three different concentrations (2%, 4%, 6%). Six different ostrich embryos were investigated in each group. First, a part of the eggshell was removed using a rotating cutter, as described before [14,23]. Then, a small 30-gauge needle connected to a plastic tube was used to puncture a vessel of the chorioallantois membrane (CAM) beneath the removed eggshell. A connection between the plastic tube and the container lid was established with an adapter (Multi-Adapter Luer, Sarstedt AG, Nurnbrecht, Germany) (Figure 2). A tube for blood tests designed for blood gas analyses and containing calcium-balanced lithium heparin (Blutgas-Monovette, SARSTEDT AG & Co. KG, Nümbrecht, Nordrhein-Westfalen, Germany) was used to draw blood samples at 1 min, 40 min, 58 min, 65 min, 90 min and 120 min after the experiment’s start. The cannula remained in the CAM vessel during the entire experiment, and patency was ensured by flushing with heparin solution. For each blood sample, we aimed to extract 500 µL of blood from the embryo. The eggs were exposed to constantly inflowing isoflurane in concentrations from 0 min to 60 min. From 61 to 120 min, constantly inflowing ambient air filled the container. In this experiment, sealing of the container (as described for oximetry, capnometry and indirect isoflurane absorption) was not performed. Blood samples were cooled to 4 °C for shipping and were analyzed in a laboratory with a focus on occupational narcotic gas exposure, e.g., in operating rooms (MVZ Medizinisches Labor Bremen GmbH, Bremen, Germany). A calibration curve was established using concentrations between 0.2 and 160 μg/mL prior to experiments. Recovery was determined at a range of 90–99%, and the lower limit of quantification was 1.0 µg/mL. For each blood sample, isoflurane was extracted at 60 °C, and headspace gas chromatography–mass spectrometry was applied using selected ion monitoring according to Kojima et al.’s description of this technique for extraction of isoflurane from human blood samples [24].

### 2.5. Statistics

Data analysis and descriptive statistics were performed using Excel (Microsoft Excel 2016, Microsoft Corporation, Redmont, WA, USA). Values were expressed as means, and standard deviations were given, if applicable.

## 3. Results

### 3.1. Oximetry and Capnometry

**Figure 3:**
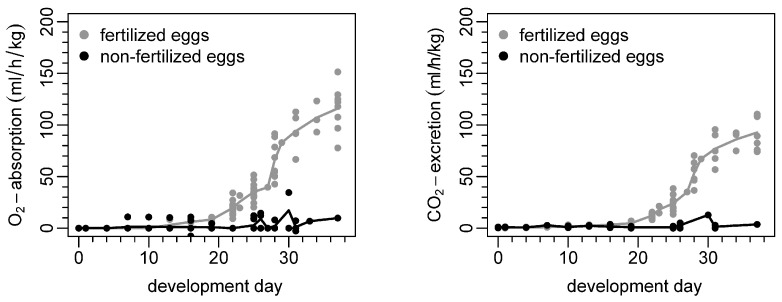
Mean oxygen consumption and mean carbon dioxide excretion during ostrich embryo development. Unit mL/h/kg was chosen according to Gefen and Ar [17].

Oxygen consumption and carbon dioxide excretion over time are shown in Figure 3 for fertilized/viable ostrich embryos and non-fertilized eggs, respectively. Differences between fertilized and non-fertilized eggs are detected from DD 19 on. Regarding fertilized eggs, the highest gas exchange rates occurred at the latest time point of this experiment, i.e., DD 37. The most pronounced increases in the gas exchange rates occurred around DDs 26–30. Non-fertilized eggs show no gas exchange, with minor artifacts/misdetection occurring after 25 days of artificial incubation.

### 3.2. Isoflurane

**Figure 4:**
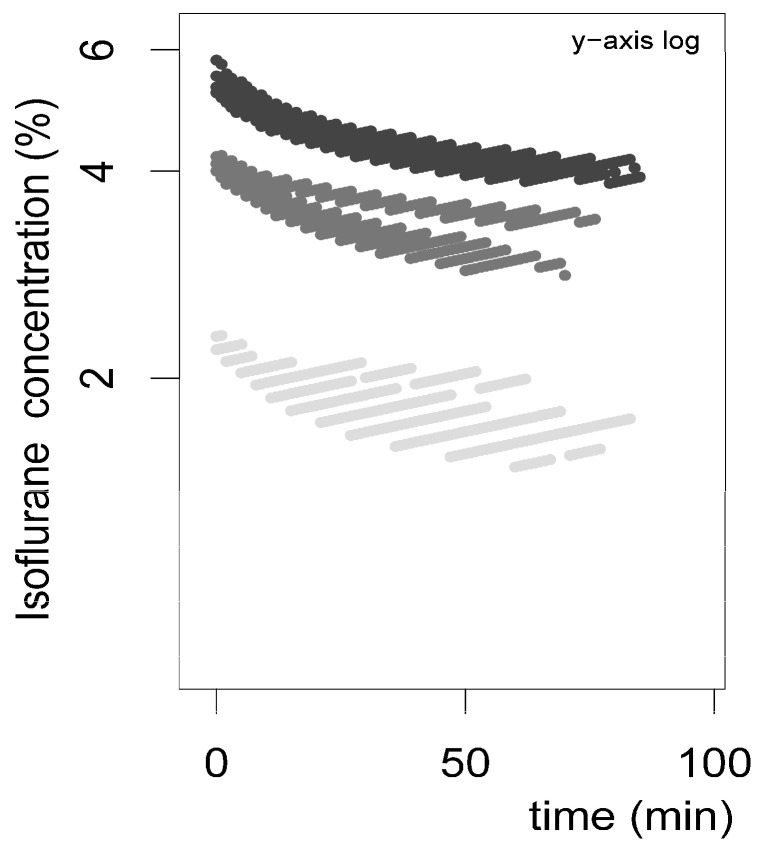
Indirect isoflurane absorption (Vol%) in the ambient gas mixture during closed-system exposure as displayed by the monitor system (mean values; 4 embryos per concentration). Measurements were recorded continuously over 100 min for different initial concentrations (6% dark gray, 4% gray, 2% light gray). Data demonstrate exponential decline patterns consistent with passive diffusion and progressive saturation of intra-egg compartments.

**Figure 5:**
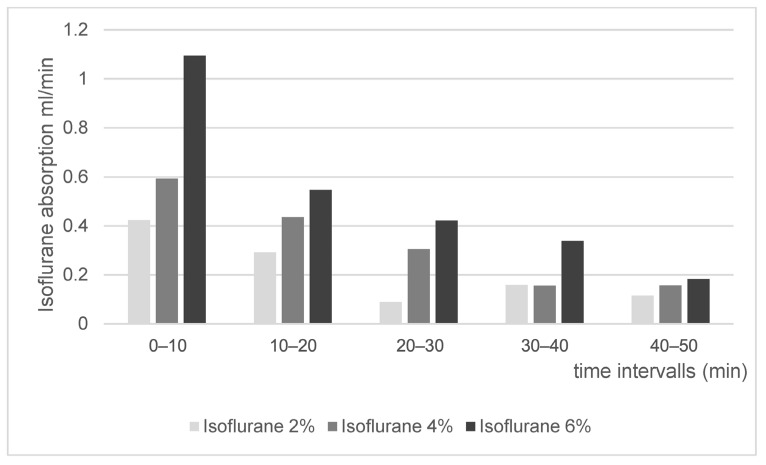
Indirect isoflurane absorption (mL/h) in the ambient gas mixture during closed-system exposure as calculated for 5 consecutive time intervals of 10 min. Unit transformation of Vol% to mL/h was calculated as described for O_2_ and CO_2_. Subsequently, the formula c(t) = c_0_ ∙ f_egg_ (t) was used; c(t) = concentration over time, c_0_ = initial concentration, f_egg_(t) = extracted fraction of isoflurane. This value was corrected for a leakage factor D (determined via closed-loop container experiments without ostrich eggs) by the formula c_residual_(t) = c(t)/(e^−D_container_∙t^).

Indirect isoflurane absorption is shown in Figure 4 and Figure 5. The gas monitoring unit displayed isoflurane content as volume percentage (Vol%) within the isoflurane–air mixture, and values were higher for the isoflurane 6% group compared to 4% and 2% (Figure 4). After calculation, absolute absorption values (mL/min) were derived, which are shown in Figure 5.

### 3.3. Blood Sampling

Data analysis was not possible for 1/18 ostrich eggs due to erroneous storage of blood tubes before shipping. Thus, 5 ostrich eggs were evaluated in the isoflurane 4% group and 6 ostrich eggs were evaluated in the isoflurane 2% and 6% groups each.

**Figure 6:**
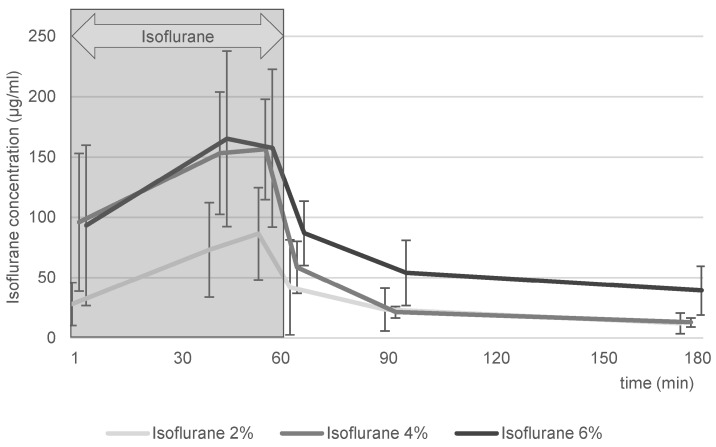
Direct quantification of isoflurane in embryonal blood samples. Blood concentrations (µg/mL) were measured over 180 min for 2%, 4%, and 6% isoflurane exposures. A rapid increase was observed during exposure, followed by a concentration-dependent decline post-exposure. Residual isoflurane remained detectable up to 120 min after cessation.

Direct isoflurane values obtained via blood samples are shown in Figure 6. The three groups, isoflurane 2%, 4% and 6%, showed a rapid increase in isoflurane serum concentration within the first three minutes, followed by a continuing increase until 40 min. Before ending isoflurane gas exposure at 60 min, serum concentrations seemed to maintain a rather stable plateau phase. Immediately after turning off the isoflurane gas and switching to regular air, isoflurane serum concentrations rapidly fell below initial values after 3 min, except for the isoflurane 2% group. After 180 min (120 min after the end of isoflurane exposure), isoflurane was still detectable in serum.

All ostrich embryos survived isoflurane exposure in a viability test 24 h after isoflurane experiments.

In general, de novo implementation for in ovo experiments using ostrich eggs requires installation of a multistage incubator (approx. $3000) and a room, as well as staff for daily egg handling during incubation (approx. 1 h/day). Ostrich eggs were purchased for $25/piece, and the cost per successful experiment (subtracting non-fertilized eggs, dead-in-shell embryos and unsuccessful vessel access) has been reported to be approx. $75 [14]. The initial and operating costs of a rodent laboratory are much higher, requiring dedicated rooms with high investment costs, as well as more frequent attendance of trained personnel (animal caretaker, veterinarian) and daily feeding of animals.

## 4. Discussion

Investigation of the underlying mechanisms of ostrich immobilization techniques is crucial for planning experiments involving dynamic PET studies for radiopharmaceuticals. The analyses described in this study go beyond what is already known from previous studies. Freesmeyer et al. and Perkas et al. described techniques using a magnetencephalography system in order to quantify ostrich embryo movement and to investigate the effects of different narcotic gases [1,13]. Effective immobilization was shown for the same concentrations of isoflurane as those used in this study; however, previous studies did not focus on the uptake mechanism and duration of narcotic gas persistence in embryonal blood.

In a step-by-step approach, the current study investigated gas uptake by ostrich embryos: First, a setup was developed using clinically available materials that were suited for anesthetic gases to avoid occupational isoflurane exposure in the staff. The container, the gas monitoring system, and the tubes were gas-tight as per manufacturers’ specifications. Nevertheless, minor leakage was detected, and thus, data correction was required for further evaluation.

### 4.1. Oximetry and Capnometry

Second, O_2_ consumption and CO_2_ excretion of ostrich eggs were determined on different DDs using the container with sealed in- and outflow. This experiment was designed to produce comparable results to Gefen and Ar, who performed similar experiments in 2001 and chose mL/h/kg as the unit of O_2_ absorption and CO_2_ excretion [17]. The current study revealed similar results, with a mean O_2_ consumption of 116 mL/h/kg and mean CO_2_ excretion of 93 mL/h/kg on DD 37, which are lower than the values described previously (O_2_ consumption of 164 mL/h/kg and CO_2_ excretion of 111 mL/h/kg). One explanation for the lower values might be differences in artificial breeding conditions, e.g., temperature, which has been described to influence energy metabolism in avian embryos during development [17]. Also, the aforementioned minor leakage could be attributed to this problem. O_2_ consumption and CO_2_ excretion show an exponential growth between DDs 19 and 31. The latest measurements were performed on DD 37, and later measurements were not obtained due to the requirement that all experiments must end before hatching. Other studies focused on later DDs and found a plateau phase without further increases in O_2_ consumption and CO_2_ excretion during the last 5 DDs. This was explained by reduced growth rates at the very end of incubation time [17,25,26]. As expected, non-fertilized ostrich eggs, which served as the control group, did not show relevant CO_2_ excretion and O_2_ absorption. Although the manufacturer states the specificity of CO_2_ measurements performed by the gas monitor system, it is assumed that interference by other carbon-containing molecules excreted by non-fertilized eggs (e.g., Methanethiol CH_3_SH, dithiol C_4_H_6_S_2_, Dimethyl sulfide (CH_3_)_2_S) is responsible for the very low CO_2_ values detected by our system (Figure 3). In summary, this experiment showed that the setup can feasibly measure gas uptake and excretion in ostrich eggs, which is a prerequisite for the following steps of isoflurane uptake quantification.

### 4.2. Isoflurane

Third, isoflurane uptake was quantified by two methods, i.e., indirect measurements determining the reduction in isoflurane in ambient air in the container and direct measurements of isoflurane in blood samples. Both experiments were performed on different ostrich eggs; therefore, there was no intraindividual comparison of indirect and direct quantification methods.

Indirect quantification results were obtained by analysis of Vol% as determined by the gas monitor system. Figure 4 shows the data over time as a scatterplot with rather large spacing, which is attributable to the scaling of the gas monitor system, which is designed for patient surveillance during anesthesia. Exhaled isoflurane concentration is sufficiently expressed in whole-number digits in patients during clinical routine. Monitor settings were not adjustable regarding the precision of values; thus, decimal values were not available. Nevertheless, isoflurane concentration showed plausible curves with gradually declining curves over time (Figure 4). Based on these values, absolute isoflurane absorption was calculated for each of the isoflurane concentration groups and was expressed as ml/min for 10 min intervals (Figure 5). This approach was deemed suitable in order to logically aggregate data, showing a higher absorption at the beginning and lower absorption at the end of isoflurane exposure, which is in line with diffusion processes. Expectedly, the isoflurane 6% group showed higher absorption values compared to the isoflurane 4% and isoflurane 2% groups.

Fourth, direct measurements of isoflurane in blood samples supported data obtained via indirect measurements described above. Blood samples taken after 1 min revealed relevant levels of isoflurane in embryonal blood, with further increases during ongoing exposure. Cessation of isoflurane inflow caused rapid decrease in isoflurane levels in embryonal blood within 3 min. Further decreases were observed up to 120 min after regular air inflow. Blood samples reveal the process of in vivo biodistribution of isoflurane concentration, which can be described as a multi-compartment model, presuming that biodistribution, metabolism and neuronal interaction in ostriches follow human patterns. The uptake of isoflurane into the egg occurs via eggshell pores by diffusion and is primarily influenced by the partial pressure gradient, the diffusion coefficient, and the barrier formed by the eggshell and its membranes. Measurements demonstrated an initially strong inward flux, which decreased as the partial pressures equilibrated; however, full equilibrium was not reached within the observation period. The blood was rapidly saturated because of its low blood–gas partition coefficient and effectively transported the anesthetic to the brain. Owing to the brain’s high perfusion, anesthetic uptake was rapidly achieved, while the diffusion of isoflurane into internal egg compartments continued. As partition coefficients have not been established for ostrich embryos, the values for humans were considered and transferred. The coefficients are blood/gas 1.4, fat tissue/gas 64 and brain/blood 1.6, which indicates fast uptake from gas to blood, as well as from blood to the brain [27,28]. This explains the very fast gas exchange during general anesthesia in humans, which is characterized by rapid compartment saturation during the distribution of gas to the blood to the brain. The rapid increases and decreases in isoflurane concentrations in ostrich embryos immediately after the start and end of isoflurane exposure matched the distribution characteristics of humans. Furthermore, the very slow equilibrium of blood/fat tissue adds to the explanation of detectable isoflurane levels even after 120 min, indicating that isoflurane, which had slowly been stored in fat tissue during exposure, was slowly released and re-entered the blood compartment. This finding is in line with data on ostrich embryo immobilization measured via magnetencephalography describing the first reappearance of embryo movements 70 min after cessation of isoflurane exposure [13]. Considering this, it can be roughly estimated that values in the blood between 50 and 100 µg/mL are needed for effective immobilization.

Data for direct comparison of ostrich embryos and isoflurane are scarce. Using chicken eggs, similar experiments have been described investigating the effect of isoflurane on small animal PET imaging [11,29]; however, these did not report isoflurane uptake quantification.

Comparison of blood levels of isoflurane in humans after surgery, in which stated levels are 0.5–1.5% (approx. 50–150 µg/mL), show that isoflurane levels in ostrich embryos are of a similar range, underlining the plausibility of the obtained results [30,31].

### 4.3. Limitations

This study has several limitations. First, the sample size of ostrich embryos (*n* = 7 for capnometry/oximetry, *n* = 4 per group for indirect isoflurane uptake, *n* = 6 per group, direct isoflurane uptake) is susceptible to outliers; thus, the data quality might be hampered. Especially regarding Figure 6 (direct isoflurane uptake), the standard deviation is rather large. However, the values match the approximate order of values described for humans; thus, plausibility can be assumed. This limitation could be addressed using a priori power calculations for future experiments. Second, drawing blood samples took a long time (mean 1.5 min/sample) due to the very small CAM vessel diameter. This could have influenced the results and presumably caused high variability (Figure 6), especially in phases with very rapid increases or decreases in isoflurane concentration in the blood. Third, analysis revealed leakage of the container, requiring data correction for leakage effects. This extra work is not necessary when precisely checking all components for gas-tightness. Another aspect that might have contributed to erroneous measurements is the segregation of isoflurane and air (isoflurane is heavier than air), leading to imprecise determination of isoflurane concentration within the container.

A general limitation of using clinical PET/CT scanners is the lower spatial resolution compared with dedicated small-animal PET systems. While clinical PET typically provide voxel sizes of approximately 1–2 mm, micro-PET systems can achieve resolutions below 0.1–0.5 mm. This difference may affect the quantitative assessment of activity distribution in small embryonic structures due to partial-volume effects; however, the relatively large size of ostrich embryos compared with typical small-animal models partly mitigates this limitation. Furthermore, when using ostrich embryos as a preliminary tool for selection of promising candidates for radiopharmaceuticals, it needs to be considered that their metabolic pathways are different. This is based on interspecies differences but also due to divergent embryonic vs. adult development stages. Limited transferability is widely accepted when performing classic animal testing using rodents. Early results have shown superior concordance between humans and ostriches vs. humans and mice in newly developed liver-directed radiopharmaceuticals [32].

## 5. Conclusions

This study investigated gas consumption in artificially incubated ostrich embryos in order to quantify processes of effective immobilization during dynamic PET studies. A setup was successfully developed, accurately measuring CO_2_ excretion and O_2_ absorption, as well as determining indirect isoflurane uptake. Finally, direct quantification of isoflurane in blood samples was obtained, with similar results for isoflurane blood concentration in ostrich embryos and humans. This study underlines the feasibility of using isoflurane to immobilize ostrich embryos.

## Data Availability

The original contributions presented in this study are included in the article. Further inquiries can be directed to the corresponding author.
